# Expert opinions in nuclear medicine: Finding the “holy grail” in infection imaging

**DOI:** 10.3389/fmed.2023.1149925

**Published:** 2023-02-27

**Authors:** Andor W. J. M. Glaudemans, Olivier Gheysens

**Affiliations:** ^1^Department of Nuclear Medicine and Molecular Imaging, University of Groningen, University Medical Center Groningen, Groningen, Netherlands; ^2^Department of Nuclear Medicine, Cliniques Universitaires Saint-Luc and Institute of Clinical and Experimental Research (IREC), Université Catholique de Louvain, Brussels, Belgium

**Keywords:** molecular imaging, infection, inflammation, PET-CT, LAFOV PET, specific radiotracers

## Abstract

Nuclear medicine imaging techniques are now widely accepted and increasingly used for diagnosing and treatment monitoring of infectious and inflammatory diseases. The latter has been exemplified by numerous recent clinical guidelines in which PET imaging is now part of the diagnostic flowcharts. In this perspective paper we discuss the current available guidelines, the current limitations, and we provide the future aims of research to achieve the holy grail in nuclear medicine: the differentiation between infection, inflammation and malignancy.

## 1. Introduction

Infectious diseases are extremely common worldwide and their incidence increases. Everyone is prone to infectious diseases, but some people more than others, including those with suppressed or compromised immune systems, young children, adults over 60 years, and those with foreign body material. Infection can be considered as the new cancer, or even worse since infectious diseases are often transmissible and drug resistant. Antimicrobial resistance has emerged as one of the leading health threats of the 21st century which requires a global and coordinated action plan to address (1).

Inflammatory diseases are not transmissible, but inflammation is part of the body’s complex biological response to external stimuli. When overacting, healthy tissues can be caught up in this reaction leading to systemic inflammation or auto-immune diseases. Furthermore, inflammation is also thought to be a key player for the development of malignancies and cardiovascular disorders.

Infectious and inflammatory diseases affect millions of people worldwide, resulting in significant morbidity and mortality and huge socio-economic burden. While still developing new drugs to treat “old” infectious diseases like HIV or tuberculosis, “new” infectious diseases are emerging and will continue to emerge, as has recently been witnessed by the COVID-19 pandemic. This illustrates in a nutshell the importance of infection and inflammation. Moreover, we have to recognize these diseases at an early stage, and diagnose them correctly in order to start as soon as possible the correct treatment and to evaluate early on treatment efficacy, all aiming at avoiding further sufferance, disability, transmission and resistance.

Diagnosis and treatment evaluation of infection and inflammation usually rely on clinical findings, laboratory parameters, and histological and/or microbiological results. The problem, however, is that clinical constitutional symptoms and laboratory parameters are often nonspecific, and that histology may not always be feasible or often requires an invasive procedure that may lead to sampling errors or complications. In addition, such assessment does not reflect the whole disease burden and activity.

Functional imaging plays a pivotal role in the diagnosis and treatment follow-up in patients with infectious or inflammatory diseases. Morphological imaging methods, such as ultrasound, computed tomography (CT) and magnetic resonance imaging (MRI) are commonly used, but focus on morphological changes and tissue distortions, which often occur at a later disease stage. In contrast, functional imaging methods, such as positron emission tomography (PET) are able to detect functional changes already at an earlier stage, and are able to evaluate treatment response after a few days or weeks. The combination of PET and CT (PET/CT) makes it possible to image functional and anatomical changes in one single imaging session.

## 2. Evidence

The most commonly used radiopharmaceutical (tracer) for this functional imaging of infectious and inflammatory diseases is [^18^F]fluorodeoxyglucose (FDG). The past decade, plenty of evidence has become available for the use of [^18^F]FDG-PET/CT in a broad range of infectious and inflammatory indications. The Inflammation & Infection Committee of the European Association of Nuclear Medicine (EANM), consisting of experts in the field, has dedicatedly worked on processing this evidence into workable diagnostic flowcharts, guidelines, and procedural recommendations for the optimum use of these nuclear medicine imaging techniques in close collaboration with and involvement of the clinical societies. This has resulted in widely adopted guidelines and recommendations for infective endocarditis (2, 3), vasculitis and polymyalgia rheumatica (4), vascular graft infections (5, 6), peripheral bone infection (7), prosthetic joint infection (8), and spondylodiscitis (9). Furthermore, [^18^F]FDG-PET/CT can be considered as an invaluable imaging tool in, e.g., patients with fever of unknown origin, both in adults and in children (10, 11), patients with bacteremia at high-risk (12), fungal infections (13, 14) and infected renal or liver cysts (15, 16).

## 3. Limitations

Although [^18^F]FDG-PET/CT has been proven a good method for several indications as shown above with acceptable diagnostic accuracy in most infectious and inflammatory diseases, it also presents several limitations. The most important one is linked to the lack of specificity of [^18^F]FDG uptake. Both infectious and inflammatory conditions demonstrate increased [^18^F]FDG uptake, making it very challenging to differentiate between infection and inflammation. In addition to immune cells, cancer cells also show enhanced [^18^F]-FDG uptake, thereby further challenging the diagnostic process. Another well-known limitation of [^18^F]FDG is the physiologic uptake throughout the body, but especially in the brain, the intestines and the urinary tract, hereby limiting the possibility to detect infection or inflammation in those organs/tissues. Thirdly, diagnosing an infection shortly after therapeutic interventions (surgery or radiation therapy) may cause difficulties, since cells involved in the wound healing process and granulation tissue also cause an increased tracer uptake. Reactive uptake due to foreign body material may occur in, e.g., vascular grafts, orthopedic implants, or prosthetic heart valves. Finally, several drugs are known to influence [^18^F]FDG uptake. Metformin increases intestinal glucose uptake, insulin (but also exercise-induced insulin secretion) results in elevated [^18^F]FDG uptake in physiological tissues (e.g., muscles), antibiotics (>7 days) are associated with a lower chance of revealing an infection focus, and corticosteroids (>10 days) reduce the sensitivity for diagnosing inflammation (e.g., vasculitis) (17).

## 4. Future

As illustrated above, [^18^F]FDG-PET/CT is an excellent tool when searching for infection or inflammation, but its non-specificity is a major drawback. Consequently, we keep searching for new radiopharmaceuticals and to obtain as much information as possible to prevent, diagnose and ideally cure these diseases. The research community continues to develop better imaging techniques and equipment, and more specific radiopharmaceuticals. We remain steadfast in our determination to achieve diagnostically accurate imaging of infection and inflammation enabling to discriminate with high precision between infection, inflammation and malignancy. We keep on looking for this “holy grail” in nuclear medicine by focusing on two major research areas to achieve this goal.

### 4.1. Toward more specific tracers

Advances in disease understanding and translational medicine have taken us to the next level of practicing medicine. Personalized medicine has come within our grasp and has the potential to provide optimized treatment based on individual patient characteristics. Oncology is leading this new way of practicing with multiple examples of clinical translation of molecular disease characteristics into personalized treatment strategies that are becoming the standard of care. In analogy to cancer, nuclear medicine techniques offer the unique opportunity to target cell subpopulations and molecules that are involved in infectious and inflammatory lesions, to select patients who will most likely benefit from a particular treatment and for early therapy follow-up. Nuclear medicine provides the opportunity to highlight the cell types and subtypes that are involved in the process, the presence of a pathogen (bacteria of fungus), or the involvement of cytokines and chemokines. Several developments are ongoing in this area, e.g., the use of radiolabeled lymphocytes and macrophages (18, 19). [^68^Ga]/[^18^F] labeled fibroblast activation protein inhibitor (FAPI), developed for oncological indications, is nowadays more and more used in inflammatory diseases for detecting active fibroblasts. Furthermore, pilot studies using specific tracers for detecting bacterial infections (20, 21) look promising. One can envision that it could become possible in the near future to tell the clinician not only that there is an infection, but also reveal the bacterial type (Gram positive or Gram negative) causing the infection, hereby guiding antibiotic treatment in an early phase.

### 4.2. Toward a higher sensitivity

The first commercial introduction of PET was in 1978, but it took another 15 years until PET really became a clinically used imaging system. The real changes occurred in the 21st century, first by introducing hybrid PET/CT camera systems, followed in 2018 by the introduction of the first digital PET/CT systems leading to important improvements in spatial resolution and signal-to-noise ratios. Despite all these advances, an average whole-body PET/CT acquisition takes around 20 min.

Another hybrid imaging technique, PET/MRI, has been introduced a couple of years ago, but did not change the nuclear medicine community as expected. At this moment, it remains more or less a research tool, with specific advantages for specific indications, but its high costs, complexity, and long scanning time remain important limitations. In the field of infection and inflammation, however, PET/MRI could be beneficial in particular cases, such as cardiac sarcoidosis and amyloidosis, inflammatory bowel diseases, diabetic foot infections and osteomyelitis, and especially in children with infection because of the significant lower radiation exposure (22).

More recently, the next game-changing technology is the large axial field of view (LAFOV) PET/CT camera, with a substantial increase in sensitivity, hereby allowing for faster scanning and/or an important reduction in injected activity, hence radiation burden (23, 24). The first LAFOV PET/CT cameras have now been installed in larger nuclear medicine departments.

For infection and inflammation imaging, these LAFOV PET/CT scanners have four major advantages:

1. LAFOV systems enable ultrafast whole-body scanning within 2–3 min compared to the standard 15–20 min acquisition on conventional PET/CT systems This is particularly beneficial for critically ill patients, patients admitted to the Intensive Care Unit, patients who suffer from pain/discomfort, or children who can now be scanned without the need for sedation (25).
2. The increased sensitivity may enable us to detect infections or inflammatory processes that were not detectable using conventional PET/CT scanners due to low metabolic activity of the disease process (chronic low-grade infections, infections with low bacterial load) or due to limited sensitivity (biofilms on prosthetic material, inflammation of cranial vessels, e.g., temporal and maxillary arteries).
3. The higher sensitivity makes it possible to scan at later time points, even after 4 or 5 half-lives of the radionuclide. This may be beneficial for some indications to have a better discrimination between perfusion/blood pool activity and inflammation, e.g., in large vessel vasculitis or cardiac sarcoidosis.
4. It is now possible to perform dynamic imaging with all the major organs in the same field of view. Since [^18^F]FDG uptake in infectious and inflammatory processes is a dynamic process, dynamic imaging may show differences in glucose metabolism kinetics between infection and inflammation, thereby hopefully allowing to differentiate between both.

While we are working on these new specific radiopharmaceuticals and evaluating all the developments in software and hardware, we still have some other issues to solve in the next years. One of them is the challenge to image the immune system, since all the cells involved in infection, inflammation and tumorigenesis are highly interconnected. Targeting one particular cell subpopulation to reach higher specificity is therefore challenging. This may be solved perhaps in the combination of radiopharmaceuticals (“cocktails”) to enhance the characterization of these complex biological processes.

Another issue may be the infrastructure of nuclear medicine departments. Having the choice of various radiopharmaceuticals to image disease processes in day-to-day practice is a great molecular weapon to personalize the patient’s management. However, this requires investments in local infrastructure and distribution facilities.

## 5. Conclusion

Nuclear medicine imaging techniques are now widely accepted and increasingly used for diagnosing and treatment monitoring of infectious and inflammatory diseases. The latter has been exemplified by numerous recent clinical guidelines in which PET imaging is now part of the diagnostic flowcharts. And the future looks bright with huge developments in both camera systems (LAFOV PET/CT) and in specific infection and inflammation tracers. In the next years, we should make use of these developments and facilitate a smooth translation in clinical practice to finally be able to differentiate between tumor and infection, between infection and inflammation, and to evaluate therapy in an early phase, aiming at a better personalized care. The race to find the holy grail of nuclear imaging in infections continues and, step by step, gets closer to the finish. However, this requires a continuing and close collaboration with clinicians and well-designed prospective studies.

## Data availability statement

The original contributions presented in the study are included in the article/supplementary material, further inquiries can be directed to the corresponding author.

## Author contributions

AG and OG contributed to the conception and design of this manuscript. AG wrote the first draft of the manuscript. All authors contributed to the article and approved the submitted version.

## Conflict of interest

The authors declare that the research was conducted in the absence of any commercial or financial relationships that could be construed as a potential conflict of interest.

## Publisher’s note

All claims expressed in this article are solely those of the authors and do not necessarily represent those of their affiliated organizations, or those of the publisher, the editors and the reviewers. Any product that may be evaluated in this article, or claim that may be made by its manufacturer, is not guaranteed or endorsed by the publisher.
